# Effectiveness of early essential newborn care on breastfeeding and maternal outcomes: a nonrandomized controlled study

**DOI:** 10.1186/s12884-022-05037-8

**Published:** 2022-09-14

**Authors:** Chuanya Huang, Lei Hu, Yonghong Wang, Biru Luo

**Affiliations:** 1 West China School of Nursing, Sichuan University/Department of Nursing, West China Second University Hospital, Sichuan University, Chengdu, 610000 China; 2 Key Laboratory of Birth Defects and Related Diseases of Women and Children, Ministry of Education, West China Second University Hospital, Sichuan University, Chengdu, 610000 China; 3 Department of Nursing, West China Second University Hospital, Sichuan University/West China School of Nursing, Sichuan University, Chengdu, 610000 China

**Keywords:** Early essential newborn care, Breastfeeding, Women, Birth, Midwifery

## Abstract

**Background:**

Breastfeeding and maternal health play crucial roles in improving newborn health, which is closely related to the development of families and society. Early essential newborn care, which emphasizes early exclusive breastfeeding and skin-to-skin contact, is recommended by the World Health Organization. This study aimed to explore the association of early essential newborn care with breastfeeding and maternal outcomes.

**Methods:**

A nonrandomized controlled study was carried out from May 2020 to January 2021 in a tertiary hospital in Chengdu city, China. Pregnant women were recruited from the maternity ward before they gave birth. Early essential newborn care was performed for 91 mother-newborn pairs after birth in the intervention group, while routine birth care was performed for 91 mother-newborn pairs in the control group. Data on breastfeeding and maternal outcomes were collected pre-test and post-test and were recorded by trained data collectors and retrieved from hospital case record files.

**Results:**

Compared with the control group, the intervention group had a higher incidence of early breastfeeding initiation, an earlier initiation and longer duration for the first breastfeeding, a higher incidence of successful first breastfeeding, more exclusive breastfeeding at hospital discharge, higher maternal breastfeeding self-efficacy, a shorter duration of the third stage of labour, lower postpartum blood loss, and lower scores of maternal pain and anxiety postpartum; the differences were statistically significant (*p* < 0.05).

**Conclusion:**

The implementation of high-quality early essential newborn care can help mothers initiate early breastfeeding, improve exclusive breastfeeding rates at hospital discharge, enhance breastfeeding self-efficacy, promote the woman’s recovery from labour, and reduce maternal anxiety and pain in the postpartum period. High-quality early essential newborn care is recommended to policymakers and medical professionals to improve breastfeeding and maternal outcomes.

**Trial registration:**

Chinese Clinical Trial Registry, Retrospective Registration (27/7/2021), registration number: ChiCTR2100049231.

## Background

Breastfeeding is the ideal method for infant feeding. It is estimated that if the breastfeeding rate were to increase to 50% worldwide, the deaths of approximately 823,000 under-five children can be avoided every year [1]. Early postnatal breastfeeding behaviour is associated with long-term breastfeeding [2]. To improve the breastfeeding rate, the World Health Organization (WHO) has recommended skin-to-skin contact between mothers and newborn infants immediately after birth and breastfeeding during the first hour after birth [3]. Studies have shown that breastfeeding within the first hour after birth can improve exclusive breastfeeding rates at 6 weeks, 10 weeks, and 6 months postpartum [4–6] and that mothers who breastfeed early have a higher acceptance of exclusive breastfeeding [7]. Compared with newborn infants who initiated breastfeeding at 2–23 h and 24–96 h after birth, newborn infants who initiated breastfeeding within the first hour after birth had lower neonatal mortality [8].

Previous studies indicated that many medical professionals, especially in the West Pacific region, often implemented outdated and harmful practices during and after birth, such as unnecessary suctioning, delayed early skin-to-skin contact between the mother and the newborn infant, as well as umbilical cord cutting immediately after birth [9]. These outdated practices lead to an increase in the risk of neonatal morbidity and mortality [10]. To improve the quality of newborn care, the *Action Plan for Healthy Newborn Infants in the Western Pacific Region (2014–2020)* was issued by the WHO Western Pacific Regional Office (WHO/WPRO) [11]. This plan aimed to give every newborn a healthy start and implement early essential newborn care (EENC) for all newborn infants. EENC contains evidence-based interventions that are simple, that are low-cost and that do not require expensive technologies. The central element of EENC is immediate skin-to-skin contact between the mother and newborn infant after birth for at least 90 min and initiation of exclusive breastfeeding when cues occur (such as drooling, tonguing, rooting, and hand biting). Additionally, midwives should appropriately delay clamping and cutting of the cord and other routine care. These practices can ensure that most newborn infants complete the first breastfeeding during the period of skin-to-skin contact and improve the early breastfeeding initiation rate, as well as strengthen the rooting reflex of the newborn infant [12]. Furthermore, implementing EENC may also have positive effects on mothers because skin-to-skin contact between mothers and newborn infants can reduce maternal pain, depression and anxiety, accelerate placental detachment, reduce postpartum haemorrhage, and promote uterine involution by promoting the secretion of oxytocin [13].

EENC was introduced to China in 2016 and had been implemented in 112 medical institutions by 2017 [14]. Yang et al. surveyed the medical institutions of four provinces that implemented EENC in China and showed that only 36.2% of the newborn infants had skin-to-skin contact with their mothers, the rate of the duration of skin-to-skin contact over 90 min was 19.7%, and the breastfeeding rate and exclusive breastfeeding rate before discharge were 76.5% and 32.2%, respectively [15]. The findings of the study by Yang et al. indicated that EENC was not fully implemented in line with the WHO guidelines in these medical institutions. Xu et al. pointed out that there were many obstacles to implementing EENC in China hospital policies, including insufficient awareness of medical professionals, shortage of human resources, and little clinical evidence about EENC in China [16]. Previous studies have explored the benefits of skin-to-skin contact and timed clamping for newborn infants separately [13, 17]. However, the EENC is an intervention package; thus, the effect of EENC should be regarded as a general effect on mothers and newborn infants. In addition, most published studies have focused on the effect of implementing EENC on improving newborn outcomes, while few studies have explored the benefits of EENC for breastfeeding and maternal outcomes. This study aimed to fill this research gap, explore the effect of implementing high-quality EENC on breastfeeding and maternal outcomes, and provide more clinical evidence for improving the health of newborn infants and mothers in the West Pacific Region.

The definitions of certain terminology used in this paper are as follows: early breastfeeding initiation, defined as the initiation of first breastfeeding within the first hour after birth; successful first breastfeeding, defined as the score of first breastfeeding assessed by the 4-item Infant Breast Feeding Assessment Tool (IBFAT) [18] is between 10 and 12; exclusive breastfeeding, defined as only breast milk given to the newborn infant without any liquid or solid food; mixed feeding, defined as breastfeeding combined with artificial feeding of the newborn infant; and artificial feeding, defined as feeding newborn infants with foods other than breast milk, such as formula milk.

## Methods

### Study design and setting

This study was a nonrandomized controlled study and was carried out from May 2020 to January 2021 in a tertiary hospital in Chengdu city, Sichuan Province, China. This hospital is one of the largest women and children’s hospitals in Sichuan Province and has two labour wards with identical health facilities and similar human resources in different hospital areas. These two wards had not implemented EENC or received any EENC coaching before this study, and they were assigned randomly to be the intervention group and control group. Each pregnant woman chose the labour ward in which she preferred to give birth at her antenatal visit in the hospital, and her selection depended entirely on her preferences. However, we began participant recruitment when the woman was awaiting delivery in the maternity ward; thus, the participants were not assigned to each group randomly.

The current study was a part of a larger trial. Because of the limitations to the length of an article, this paper focuses only on breastfeeding and maternal outcomes.

### Participants

Participants in this study comprised women and their newborn infants. Pregnant women were recruited from the maternity ward when they were admitted to hospitals for await delivery and with no signs of labour. Pregnant women who met the following inclusion criteria were considered eligible and were invited by the researchers to participate in this study: (1) aged over 18 years, (2) gestational age between 37 and 42 weeks, (3) singleton pregnancy, (4) vaginal delivery, (5) no severe pregnancy complications and/or underlying disease, and (6) no medical indications against breastfeeding. If the woman was transferred from vaginal delivery to caesarean section or the newborn infant had an abnormal birthweight (< 2500 g or > 4000 g), deformities or needed to be transferred to the neonatal intensive care unit (NICU) immediately after birth, the mother and infant were excluded from the study. Written informed consent was obtained from all participants. Ethical approval was received from the hospital ethics review board.

### Interventions

Participants in the intervention group received the EENC interventions after birth from midwives in the intervention group, while participants in the control group received routine birth care from midwives in the control group. These interventions delivered in the delivery room. The midwives in the intervention group received 5-month training sessions from national and provincial facilitators, following the guidelines formulated by the WHO [19]. After 5-month training sessions, a pilot study on 18 mother-newborn pairs was conducted in October 2020 to ensure that every midwife could implement the EENC correctly. The formal interventions were performed from November 2020 to January 2021.

EENC interventions include (1) drying the newborn infant immediately and thoroughly within five seconds after birth, (2) immediate skin-to-skin contact within the first minute and lasting for at least 90 min, (3) exclusive breastfeeding, (4) appropriately timed clamping and cutting of the cord, and (5) other routine care – eye care, vitamin K, immunizations, weighing and examinations [11]. The duration of implementing EENC was between 90 to 120 min.

The sequence of routine birth care in this hospital was (1) drying of the newborn infant, (2) placement of the newborn infant on a heated table to keep warm for 20 min, during which the umbilical cord is clamped and the weight and length are measured, (3) vaccination, (4) skin-to-skin contact between the mother and newborn infant, and (5) exclusive breastfeeding after the third stage of labour. The duration of implementing routine birth care was between 90 to 120 min.

For both groups, the same postnatal care and education were delivered to mothers by midwives, including the contents of breastfeeding, diet, physical activity, safety, urine output, and stool output.

### Objectives

This study aimed to explore the effect of implementing EENC on breastfeeding and maternal outcomes. We hypothesized that implementing EENC could improve the breastfeeding outcomes and help mothers recover from delivery, especially for the incidence of early breastfeeding initiation.

### Measures and data collection

Variables collected at baseline for women included age, educational level, height, weight, gestational age, previous obstetric history, anxiety, and nipple pattern. Variables for newborn infants included sex, length, and birthweight. Among these variables, the anxiety of women was assessed by the Chinese version of the strait form of the State-Trait Anxiety Inventory (STAI-S), which was developed by Spielberger in 1970 and was introduced to China in 1988 [20]. The STAI-S has 20 self-report items and items are scored on a four-point Likert scale of 1 (not at all) to 4 (severe), with the scores summated to derive a total score ranging from 20 to 40 points. The Cronbach’s α of the Chinese version of STAI-S was 0.91. Higher STAI-S scores indicate severer anxiety. In addition, the nipple pattern was classified into three types, namely, normal, flat, and inverted patterns, and assessed by two female data collectors.

The primary outcome of the current study was the incidence of early breastfeeding initiation. If the first breastfeeding was initiated successfully within the first hour after birth, the early breastfeeding initiation was considered and would be recorded by the data collectors.

The second outcome of this study consisted of some breastfeeding-related outcomes and maternal outcomes; namely, the time of rooting reflex occurrence, the initiation time and duration of first breastfeeding, the number of successful first breastfeeding, the time when formula milk is first served, the total amount of formula milk given before discharge, the number of breastfeeding within the first day after birth, the feeding pattern before discharge, the duration of the third stage of labour, the postpartum blood loss within 2 h after birth, and the pain and anxiety of the woman after birth. Data on the duration of the third stage of labour and the postpartum blood loss within 2 h after birth were retrieved from hospital case record files. The woman’s pain was evaluated by means of the Visual Analogue Scale (VAS) [21] at 30 min, 60 min, and 120 min after birth. Anxiety was evaluated by the state form of the State-Trait Anxiety Inventory (STAI-S) [22] at 120 min after birth. Other variables were recorded by data collectors. Additionally, the 4-item Infant Breast Feeding Assessment Tool (IBFAT) [18] was used to assess the success of the first breastfeeding by data collectors. The total score of IBFAT ranges from 0 to 12, with 10–12 being the scores for vigorous and effective breastfeeding. The Breastfeeding Self-efficacy Scale Short Form (BSES-SF) [23] was used to assess the confidence of women to breastfeed before discharge from the hospital, with a higher BSES-SE score indicating stronger breastfeeding self-efficacy.

The data collectors were all women with medical educational background. Before the study, data collectors received the methods for collecting data by researchers. They were permitted to enter the ward to collect data by both the participants and the ethnic committee of hospitals.

### Sample size

PASS version 15.0 was used to calculate the sample size. We estimated the sample size based on the primary outcome of this study, which is the incidence of early breastfeeding initiation. The results of the pilot study showed that the incidence of early breastfeeding initiation were 77.8% and 44.4% in the intervention group and the control group, respectively. Hence, a sample size of 100 participants would be required (*α* = 0.05, *β* = 0.1) [10]. Considering that the drop-out rate was 10%, the minimum sample size needed was 110 participants, with 55 participants in each group. To reduce sampling error [24], we include all pregnant women who met the inclusion criteria in the study during the recruitment phase.

### Blinding

The current study was a single-blinded trial. It was impossible to blind the midwives and data collectors in the delivery rooms because midwives were responsible for implementing EENC or routine birth care and data collectors were responsible for assessing and recording. Hence, only participants were blinded.

### Statistical methods

SPSS version 25.0 was used to analyse the data. The smallest unit that is being analyzed to assess intervention effects was the group. The mean ± standard deviation (SD) and median (interquartile range, IQR) were used to describe continuous data, and t test and Mann–Whitney U test were used to identify the differences. The number (n) and percentage (%) were used to describe categorical data, and the chi-square test and Fisher’s exact test were used to identify differences.

## Results

### Baseline information of participants

**Fig. 1:**
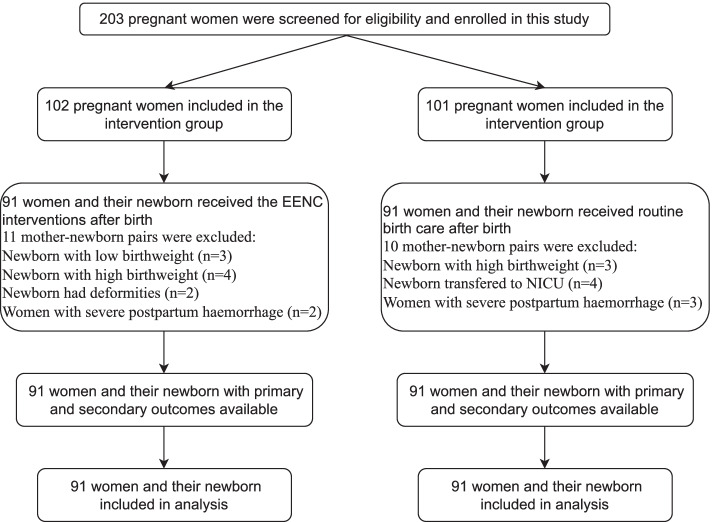
Flow of participants through each stage of the study

In total, 203 pregnant women were recruited for this study from November 2020 to January 2021, with 102 included in the intervention group and 101 in the control group. Figure 1 shows the flow of participants through each stage of the study. Ultimately, there were 91 mother-newborn pairs in the intervention group and 91 mother-newborn pairs in the control group. Table 1 shows the basic information of all participants. There were no significant differences between the two groups regarding the baseline information.

**Table 1:** Baseline information of participants

Variables	Intervention group (*n* = 91)	Control group (*n* = 91)	*p*^a^
**Maternal**			
Age (years), Mean ± SD	30.23 ± 2.78	30.32 ± 3.27	0.845
Educational level (%)			0.073
High school and below	2 (2.2)	8 (8.8)	
Junior college	14 (15.4)	8 (8.8)	
Undergraduate and above	75 (82.4)	75 (82.4)	
Gestational age (weeks), Mean ± SD	39.63 ± 0.98	39.51 ± 0.97	0.403
Previous obstetric history, n (%)			0.628
Primiparous	65 (71.4)	62 (68.1)	
Multiparous	26 (28.6)	29 (31.9)	
Nipple pattern of women, n (%)			0.415
Raised	77 (84.6)	83 (91.2)	
Flat	10 (11.0)	6 (6.6)	
Sunken	4 (4.4)	2 (2.2)	
STAI-S^*^, Mean ± SD	42.92 ± 9.45	43.52 ± 10.15	0.684
Height (m^*^), Mean ± SD	1.60 ± 0.04	1.61 ± 0.05	0.107
Weight (kg^*^), Mean ± SD	64.69 ± 7.82	66.50 ± 7.03	0.102
**Newborn**			
Sex, n (%)			0.767
Male	47 (51.6)	45 (49.5)	
Female	44 (48.4)	46 (50.5)	
Length (cm^*^), Mean ± SD	49.78 ± 1.58	50.04 ± 1.48	0.245
Birthweight (g^*^), Mean ± SD	3251.65 ± 362.22	3232.53 ± 317.33	0.705

^a^ the two-tailed t test or chi-square test were used for comparisons between the intervention group and the control group

^*^STAI-S the state form of the State-Trait Anxiety Inventory, *m* metres, *kg* kilograms

### Breastfeeding within 2 h after birth in the two groups

The incidence of early breastfeeding initiation in the intervention group was higher than that in the control group (*n* = 69 vs. n = 39, *p* < 0.001). The first breastfeeding in the intervention group started earlier (48.02 ± 16.30 min vs. 66.97 ± 35.41 min, *p* < 0.001) and lasted longer (34.98 ± 15.02 min vs. 22.30 ± 11.70 min, *p* < 0.001) than that in the control group. Additionally, the mean IBFAT scores of the first breastfeeding were higher (10.05 ± 2.17 vs. 8.68 ± 2.04, *p* < 0.001). Furthermore, more successful first breastfeeding (*n* = 83 vs. *n* = 68, *p* = 0.003) were observed in the intervention group. However, there was no significant difference in the time of rooting reflex occurrence. (Table 2).

**Table 2:** Breastfeeding within 2 h after birth in the two groups

	Intervention group (*n* = 91)	Control group (*n* = 91)	*p*^a^
Early breastfeeding initiation, n (%)	69 (75.8)	39 (42.9)	< 0.001
Occurrence of rooting reflex (min), median (IQR)	18 (12, 22)	19 (14, 29)	0.076
Successful first breastfeeding, n (%)	83 (91.2)	68 (74.7)	0.003
Initiation of first breastfeeding (min), mean ± SD	48.02 ± 16.30	66.97 ± 35.41	< 0.001
IBFAT of first breastfeeding^*^, Mean ± SD	10.05 ± 2.17	8.68 ± 2.04	< 0.001
Duration of first breastfeeding (min), mean ± SD	34.98 ± 15.02	22.30 ± 11.70	< 0.001

^a^ the two-tailed t test, Mann–Whitney U test, or chi-square test was used for comparisons between the intervention group and the control group

^*^IBFAT Infant Breast Feeding Assessment Tool, *min* minute

### Breastfeeding before discharge in the two groups

The median time at which the formula milk was first served in the intervention group was later than that in the control group (4 h vs. 2 h, *p* < 0.001), and the median amount of formula milk given to babies before discharge was higher in the control group than in the intervention group (70 ml vs. 90 ml, *p* < 0.001). The number of breastfeeding within 24 h after birth in the intervention group was greater than that in the control group (*n* = 7 vs. *n* = 5, *p* < 0.001). Regarding the feeding pattern, the number of exclusive breastfeeding in the intervention group was greater than that in the control group (*n* = 67 vs. *n* = 40), with less mixed breastfeeding (*n* = 24 vs. *n* = 47) and artificial breastfeeding (*n* = 0 vs. *n* = 4). The women in the intervention group had higher breastfeeding self-efficacy assessed by the BSES-SF (55.78 ± 8.96 vs. 46.74 ± 10.08, *p* = 0.024). (Table 3).

**Table 3:** Breastfeeding before discharge in the two groups

	Intervention group (*n* = 91)	Control group (*n* = 91)	*p*^a^
The time when formula milk is first served, (h^*^), median (IQR)	4 (2, 6)	2 (1, 4)	< 0.001
Amount of formula milk given before discharge (ml^*^), median (IQR)	70 (45, 127.5)	90 (65, 150)	< 0.001
Number of breastfeeding within 24 h after birth, n (%)	7 (6, 8)	5 (4, 6)	< 0.001
Feeding pattern before discharge, n (%)			< 0.001
Exclusive breastfeeding	67 (73.6)	40 (44.0)	
Mixed feeding	24 (26.4)	47 (51.6)	
Artificial feeding	0	4 (4.4)	
BSES-SF before discharge^*^, mean ± SD	55.78 ± 8.96	46.74 ± 10.08	< 0.001

^a^ the two-tailed t test, Mann–Whitney U test, or Fisher’s exact test were used for comparisons between the intervention group and the control group

^*^BSES-SF Breastfeeding Self-efficacy Scale Short Form, *h* hour, *ml* millilitre, *g* gram

### Duration of third stage of labour, postpartum blood loss, pain and anxiety of women

Compared to those in the control group, the duration of the third stage of labour was shorter (5.25 ± 5.66 min vs. 6.10 ± 2.92 min, *p* < 0.001), and the amount of postpartum blood loss within 2 h after birth was lower (234.64 ± 63.65 ml vs. 281.37 ± 72.29 ml, *p* < 0.001) in the intervention group. The mean VAS (at 30 min, 1 h, and 2 h) and STAI-S scores in the control group were higher than those in the intervention group, which indicated that pain and anxiety were more severe in the control group (Table 4).

**Table 4:** The duration of the third stage of labour, postpartum blood loss, pain, and anxiety after birth between the two groups

	Intervention group (*n* = 91) Mean ± SD	Control group (*n* = 91) Mean ± SD	*p*^*a*^
Duration of the third stage of labour (min), mean ± SD	5.25 ± 5.66	6.10 ± 2.92	< 0.001
Postpartum blood loss within 2 h after birth (ml), mean ± SD	234.64 ± 63.65	281.37 ± 72.29	< 0.001
VAS at 30 min, mean ± SD	1.73 ± 0.94	2.64 ± 1.67	< 0.001
VAS at the first hour, mean ± SD	0.79 ± 0.88	1.46 ± 1.34	< 0.001
VAS at the second hour, mean ± SD	0.41 ± 0.76	0.79 ± 0.91	0.002
STAI-S^*^, mean ± SD	25.62 ± 3.88	29.35 ± 5.88	< 0.001

^a^ the two-tailed t test was used for comparisons between the intervention group and the control group

^*^VAS Visual Analogue Scale, *STAI-S* the state form of State-Trait Anxiety Inventory, *min* minute, *ml* millilitre

## Discussion

This study compared the effect of EENC and routine birth care on breastfeeding and maternal outcomes in a tertiary hospital in China. The results showed that EENC can improve the early breastfeeding initiation, establish correct breastfeeding behaviour, increase the self-efficacy in breastfeeding among mothers and help them recover from childbirth.

Although there were more primiparous women in the intervention group, our results showed that the breastfeeding outcomes in the intervention group were better than that in the control group. Some studies showed that women who have breastfed previously have better breastfeeding outcomes than primiparous women [25, 26]. However, the study by Anette et al. showed that parity cannot affect the duration of exclusive breastfeeding or any breastfeeding, but early first breastfeeding can lead to a positive impact [27]. Similarly, previous studies also pointed out that although inverted or flat nipples would hinder breastfeeding [28], if the babies can be breastfed early, they are more likely to attach and can be fed well in the postnatal period. In this study, although more women with inverted and flat nipples were in the intervention group, the breastfeeding outcomes were still better than that in the control group. For primiparous women and women with flatted or inverted nipples, EENC may therefore also be recommended as it can improve breastfeeding outcomes.

The findings of this study indicated that the intervention group had a higher incidence of early breastfeeding initiation, earlier initiation and longer duration of first breastfeeding, and a higher IBFAT score for first breastfeeding. Similar findings have been reported in studies by Aiping G et al. and Min et al. [12, 29]. Early breastfeeding is an important factor for constructing correct breastfeeding behaviour. The WHO proposed *Protecting, promoting and supporting breastfeeding in facilities providing maternity and newborn services* in 2017 [30], which emphasized that medical institutions should provide all feasible support to help women initiate early breastfeeding. Previous studies also showed that women who initiated breastfeeding within the first hour after birth had a higher acceptance of breastfeeding, which is especially important for improving the exclusive breastfeeding rates up to 6 months postpartum [5, 6]. EENC interventions contain a long duration of skin-to-skin contact, which is a key factor in ensuring the success of early breastfeeding. The study by Mahmood et al. showed that newborn infants with successful skin-to-skin contact can initiate first breastfeeding 62 min earlier than newborn infants with routine birth care, and the success rate of first breastfeeding increased by 26.3% [31]. In addition, EENC recommends that midwives assist women in initiating first breastfeeding when the newborn infants experience a rooting reflex and active breast-seeking action, which is in line with newborn infants’ instincts and can avoid excessive intervention in breastfeeding. Therefore, implementing EENC could increase the rate of early breastfeeding initiation and successful first breastfeeding.

In the current study, the rates of exclusive breastfeeding and the breastfeeding self-efficacy of women at hospital discharge in the intervention group were higher than those in the control group, which were impacted mainly by the early skin-to-skin contact between mothers and newborn infants and successful first breastfeeding. The study by Almqvist et al. showed that the issues of breastfeeding encountered by women in the early postpartum period were the main reason they gave up exclusive breastfeeding [32]. Success in the first breastfeeding means that the issues in the process of breastfeeding will be partly solved with the help of health professionals. Therefore, the women in the intervention group could gain confidence and skills from the experience of success in the first breastfeeding, which would in turn motivate them to perform exclusive breastfeeding [33]. The WHO recommends that unless there are medical indications, the staff of medical institutions should dissuade women and their families from providing any food other than breast milk to their infants. However, in the clinical practice setting, the phenomenon of mothers or family members feeding infants with formula milk or other food is unavoidable even though breast milk is sufficient because midwives and nurses cannot help every woman solve the problems encountered in breastfeeding due to the demands of their work. The study by Raghavan et al. showed that formula milk given to babies on the first day emerged as the only independent predictor of failure to continue exclusive breastfeeding at 6 weeks after birth (OR 2.96; 95% CI 1.09–8.06) [5]. In this study, newborn infants in the intervention group were given formula milk for the first time approximately 2 h later than those in the control group, and the total amount of formula milk added before discharge was also lower, which indicated that the construction of correct breastfeeding behaviour within the first hour after birth can reduce the use of unnecessary formula milk to some extent. Additionally, babies in the intervention group had more breastfeeding times within 24 h postpartum on the first day postpartum than those in the control group, indicating that the implementation of EENC can help women breastfeed correctly and have higher breastfeeding self-efficacy, which is conducive to the growth and development of newborn infants [34].

Our findings also showed that the EENC can help women recover from labour. The women in the intervention group had a shorter duration of the third stage of labour and lower postpartum blood loss, which is in line with the study by Yuan et al. [35]. During skin-to-skin contact, sucking from newborn infants can stimulate the nerve endings of the maternal nipple and then promote the synthesis and secretion of oxytocin [36]. Oxytocin can stimulate uterine contraction directly, reduce the interference of oxidative stress on uterine contraction, and finally reduce postpartum blood loss [37, 38]. In addition, placing the newborn infant on the mother’s breast and abdomen plays a similar role to massage, which can also promote the contraction of the uterus [13]. Furthermore, lower levels of postnatal anxiety and pain among mothers were observed in the intervention group, which may be related to the secretion of oxytocin and the joy of successful breastfeeding. Previous studies indicated that oxytocin can increase the threshold of maternal pain perception [39] and alleviate maternal anxiety [40, 41].

This study systematically explored the effects of EENC on breastfeeding and maternal outcomes and provided more evidence for the implementation of EENC in the future. However, this study also has some shortcomings. First, the design of this study is quasi-experimental. Due to hospital policies and funding limits, the participants could not be randomly assigned to two groups. However, because the intervention and control measures are implemented in two wards of the same hospital, which have similar human resources and facilities and are far away from each other, contamination and bias were excluded as much as possible. Second, the results of pain and anxiety were self-reported variables, so self-report bias cannot be avoided. Third, follow-up in this study lasted until the mother was discharged from the hospital, so a longer-term follow-up study to clarify the long-term effect can be considered in the future. Last, although the sample size had been previously calculated, this study was conducted only in a tertiary hospital, so the generalization of the results is limited. Large-sample and multicentre randomized controlled trials are necessary to further clarify the effect of EENC.

## Conclusion

The implementation of EENC is associated with better breastfeeding and maternal outcomes, which can not only improve the early initiation of breastfeeding and exclusive breastfeeding rate but also relieve the anxiety and pain of the mother and increase her confidence in breastfeeding at hospital discharge. Hence, it is strongly recommended that policymakers and medical professionals implement EENC in clinical practice to improve the outcomes of both women and infants.

## Data Availability

All raw data generated or analyzed during this study are available from the corresponding author upon reasonable request.

## Abbreviations

EENC: Early Essential Newborn Care
IQR: Inter Quartile Range
WHO: World Health Organization
SD: Standard deviation
NICU: Neonatal intensive care unit
WPRO: Western Pacific Regional Office
OR: Odds ratio
CI: Confidence interval
IBFAT: Infant Breast Feeding Assessment Tool
BSES-SF: Breastfeeding Self-efficacy Scale Short Form
VAS: Visual Analogue Scale
STAI-S: The state form of State-Trait Anxiety Inventory
